# Identification and validation of a nomogram for psoriasis diagnosis: a novel biomarker combination with stable high expression in the early stage

**DOI:** 10.3389/fmed.2026.1798926

**Published:** 2026-04-21

**Authors:** Juanli Liu, Qingqing Hu, Qianjin Zhang, Renshan Zheng

**Affiliations:** Binhai County People’s Hospital, Yancheng, Jiangsu, China

**Keywords:** AGP, nomogram, ORM1, ORM2, psoriasis

## Abstract

**Objective:**

Psoriasis is a complex chronic inflammatory skin disease. While typical cases are often diagnosed based on clinical features, achieving objective assessment of disease activity and precise early-stage identification remains a clinical focus. This study aims to evaluate the diagnostic value of monocytes (MONO), Orosomucoid-1 (ORM1), Orosomucoid-2 (ORM2), and Alpha-1-acid glycoprotein (AGP), and to develop a nomogram for the objective and early identification of psoriasis.

**Methods:**

This retrospective case-control study included 140 participants, comprising 70 patients with psoriasis confirmed by the dermatology department and 70 healthy individuals who underwent routine health examinations during the same period. Demographic, clinical, and laboratory data (including MONO, ORM1, ORM2, and AGP) were collected for all participants. Potential risk factors were initially screened using univariate Logistic regression, followed by feature selection via the combination of Lasso regression and Boruta algorithm to identify features with the highest predictive value. Restricted cubic spline (RCS) plots were utilized to visually illustrate the non-linear associations between the selected variables and the risk of psoriasis onset. Finally, the selected features were incorporated into a multivariate Logistic regression model to determine the independent risk factors, and a diagnostic nomogram was constructed accordingly.

**Results:**

Based on the results of univariate analysis, LASSO regression and Boruta algorithm, we ultimately selected four key variables, namely MONO, ORM1, ORM2 and AGP, for the construction of the subsequent multivariate diagnostic model. The calibration curve of the model showed that the actual probability was highly consistent with the predicted probability, indicating that the model had good calibration performance. The receiver operating characteristic (ROC) curve indicated that the overall predictive ability of the model was excellent, with an area under curve (AUC) of 0.888 (95% CI: 0.835–0.941). In addition, the ROC curves of each variable analyzed separately showed that ORM2 (AUC = 0.777), ORM1 (AUC = 0.720), MONO (AUC = 0.638) and AGP (AUC = 0.673) all had different degrees of predictive ability for the risk of psoriasis.

**Conclusion:**

The novel biomarker combinations with stable and high expression in the early stage have shown great potential in the research of psoriasis. These research results should be applied to clinical.

## 1 Introduction

Psoriasis is a complex immune-mediated skin disease characterized by red patches and silvery scales, which significantly affects the quality of life of patients. Studies show that the incidence of psoriasis is higher in high-income countries and among the elderly, while it is relatively lower in low-income countries (1). For instance, the prevalence of psoriasis in some European countries can be as high as 3–5% (2). The diagnosis of psoriasis is primarily clinical, relying on characteristic skin lesions and medical history, which is generally straightforward in typical cases. The typical symptoms of psoriasis include the appearance of red patches on the skin, which are covered with silver-white scales. These patches are commonly found on areas such as elbows, knees, scalp, and lower back (3). Despite the reliability of clinical diagnosis, there is a lack of sensitive and objective biomarkers to quantify systemic inflammation and monitor disease progression, particularly in the subclinical or early stages (4).

The pathogenesis of psoriasis is driven by a systemic inflammatory milieu where various inflammatory factors and immune cells play crucial roles (5). Studies have shown that the MONO of patients with psoriasis exhibit abnormalities in both quantity and function, especially under inflammatory conditions, where their activation state and cytokine secretion capacity increase, which may be closely related to the pathogenesis of psoriasis (6). AGP is an important acute-phase reaction protein, mainly synthesized by the liver and playing a crucial role in various physiological and pathological conditions. Studies have shown that the glycosylation state of AGP is closely related to its biological functions. Abnormal glycosylation can lead to the occurrence of various diseases, including cancer and autoimmune disorders (7). The research has found that the serum AGP levels of patients with psoriasis are positively correlated with the severity and activity of the disease. Especially during the acute attack period, the concentration of AGP significantly increases. This increase may be related to the inflammatory response and immune activation of psoriasis, reflecting the potential of AGP as an inflammatory marker (8). Studies have shown that ORM1 increases as the severity of psoriasis rises, and it can serve as a real-time indicator of “inflammatory activity” (9). The high expression of ORM2 is positively correlated with the severity of psoriasis, and its level is associated with the Psoriasis Area Severity Index (PASI) score. This suggests that ORM2 may serve as a biomarker for psoriasis, allowing for the assessment of disease activity and treatment response (10).

The monocyte-to-lymphocyte ratio (MLR), as a new inflammatory marker, has gradually gained attention in clinical applications of various diseases in recent years (11). The changes in MLR are closely related to the activity level of psoriasis. An increase in MLR usually indicates a worsening of the disease, while a decrease in MLR after treatment may reflect an improvement in the condition (12). SII and SIRI have a good predictive ability in patients with psoriasis, which can assist clinicians in evaluating the prognosis of patients and formulating appropriate treatment strategies (13). Both were positively correlated with the PASI score and could independently predict disease activation and the efficacy of biologic agents (14).

In the management of psoriasis, objective monitoring and timely intervention are crucial for improving patient outcomes. Through the research on biomarkers such as MONO, ORM1, ORM2, and AGP, new tools can be provided for the objective identification and activity assessment of psoriasis.

## 2 Materials and methods

### 2.1 Study population

This study was conducted at Binhai County People’s Hospital from January 2024 to October 2025, involving a total of 140 subjects. Among them, 70 patients diagnosed with psoriasis in the dermatology department (the study group) and 70 individuals who underwent health check-ups during the same period (the control group) were included. This research protocol has been approved by the Ethics Committee of Binhai County People’s Hospital (Approval Number: 2025BYKYLL043). All procedures were strictly carried out in accordance with the principles of the 2013 Revised Helsinki Declaration.

Inclusion criteria: (1) Age ≥ 18 years; (2) Diagnosed with psoriasis vulgaris based on clinical features or histopathological examination; (3) Complete clinical data and good compliance, able to cooperate to complete the Psoriasis Area and Severity Index (PASI) score; (4) Voluntarily participated in this study and signed the informed consent form.

Exclusion criteria: (1) Received systemic treatment (such as biologics, immunosuppressants, systemic retinoid drugs, etc.) within 3 months prior to enrollment; (2) Received phototherapy within 1 month prior to enrollment; (3) Used topical anti-psoriasis drugs (such as glucocorticoids, retinoid drugs, etc.) within 2 weeks prior to enrollment; (4) Had other autoimmune or inflammatory diseases (such as rheumatoid arthritis, systemic lupus erythematosus, inflammatory bowel disease, etc.); (5) Had a clear history of bacterial, viral or fungal infection within 2–4 weeks prior to enrollment (such as upper respiratory tract infection, pneumonia, etc.); (6) Had a history of or was currently suffering from malignant tumors; (7) Had severe dysfunction of organs such as the heart, liver, or kidneys; (8) Were pregnant or breastfeeding.

### 2.2 Collection of clinical data

Collect all the clinical baseline data of the subjects, including: (1) demographic characteristics: age, gender, smoking history and drinking history; (2) laboratory indicators: white blood cell count (WBC), hemoglobin (Hb), neutrophil count (NEU), lymphocyte count (LYM), monocyte count (MONO) and platelet count (PLT), etc.; (3) underlying diseases: past medical history (such as hypertension, diabetes, etc.). All laboratory indicators were collected by experienced laboratory physicians and tested using a standardized fully automated biochemical analyzer; past medical history and personal information were verified and recorded by specialist physicians through questioning.

### 2.3 Calculation of inflammatory index

Neutrophil-to-Lymphocyte Ratio (NLR) = NEU/LYM;

Lymphocyte-to-Monocyte Ratio (LMR) = LYM/MONO;

Monocyte-to-Lymphocyte Ratio (MLR) = MONO/LYM;

Platelet-to-Lymphocyte Ratio (PLR) = PLT/LYM;

Systemic Immune-Inflammation Index (SII) = NEU × PLT/LYM;

Systemic Inflammatory Response Index (SIRI) = NEU × MONO/LYM.

### 2.4 Determination of solubility of orosomucoid-1 (ORM1), orosomucoid-2 (ORM2) and alpha-1-acid glycoprotein (AGP)

All the subjects had their peripheral venous blood samples collected within 1 h after the visit. The samples were immediately sent to the Central Laboratory of Binhai County People’s Hospital for processing, where the serum was collected and then stored at −80 ° in a refrigerator for unified testing. The concentrations of ORM1, ORM2 and AGP in the serum were quantitatively detected using the Enzyme-Linked Immunosorbent Assay (ELISA) method. The operation steps were carried out strictly in accordance with the instructions of the reagent kit.

### 2.5 PASI score classification

Two experienced dermatologists independently evaluated the conditions of patients with psoriasis using the PASI scoring method. The scoring criteria covered four aspects: lesion area, erythema, infiltration/hyperplasia, and scales. The total score ranged from 0 to 72. According to the internationally recognized severity grading standard, the patients were divided into two groups: the mild group (PASI < 10 points) and the moderate-to-severe group (PASI greater than or equal to 10 points) (15, 16).

### 2.6 Statistical analysis

The baseline characteristics of all included patients were stratified according to whether they had developed psoriasis or not. Variables that did not follow a normal distribution were expressed as interquartile ranges, and differences between groups were compared using the Mann–Whitney U test. Categorical variables were expressed as percentages and compared using the chi-square test. First, potential risk factors were screened using univariate Logistic regression (variables with *p* < 0.2 were retained). Subsequently, feature selection was conducted by combining Lasso regression and Boruta algorithm. Redundant variables were removed through compressing regression coefficients and using the random forest algorithm, and the most predictive features were selected. The non-linear relationship between the screening variables and the risk of psoriasis was visually presented using the restricted cubic spline (RCS) plot. Finally, the selected features were incorporated into the multivariate Logistic regression model to determine the independent risk factors for psoriasis and a nomogram prediction model was constructed. The discrimination and calibration of the model were evaluated using indicators such as the receiver operating characteristic (ROC) curve and calibration curve. All statistical analyses were conducted using R software (version 4.3.0) and STATA 17.0 (64-bit), with a two-sided *p*-value < 0.05 considered statistically significant.

## 3 Results

### 3.1 Baseline characteristics of the study subjects included

This study included a total of 140 subjects (70 in the psoriasis group and 70 in the healthy control group) (Table 1). The analysis of demographic and clinical basic characteristics revealed that the two groups were well balanced: there were no statistically significant differences in age, gender, smoking history, drinking history, and the prevalence rates of common comorbidities (hypertension, diabetes) (all *p* > 0.05).

**TABLE 1:** Baseline characteristics of the included population.

Variables	Total (*N* = 140)	Psoriasis	Non-psoriasis	*P*-value
		**(*N* = 70)**	**(*N* = 70)**	
Demographics				
Age, years	48.00 (34.25, 58.00)	47.00 (35.75, 58.00)	50.50 (32.75, 56.50)	0.869
Gender, n(%)				0.233
Female	79 (56.4%)	43 (61.4%)	36 (51.4%)	
Male	61 (43.6%)	27 (38.6%)	34 (48.6%)	
Alcohol, n(%)				0.850
No	101 (72.1%)	50 (71.4%)	51 (72.9%)	
Yes	39 (27.9%)	20 (28.6%)	19 (27.1%)	
Smoking, n(%)				0.466
No	96 (68.6%)	46 (65.7%)	50 (71.4%)	
Yes	44 (31.4%)	24 (34.3%)	20 (28.6%)	
Previous illness				
Hypertension, n(%)	112 (80.0%)	57 (81.4%)	55 (78.6%)	0.673
No				
Yes	28 (20.0%)	13 (18.6%)	15 (21.4%)	
Diabetes, n(%)	124 (88.6%)	64 (91.4%)	60 (85.7%)	0.288
No				
Yes	16 (11.4%)	6 (8.6%)	10 (14.3%)	
Laboratory parameters				
WBC 10^∧^9/L	6.09 (5.23, 7.50)	6.46 (5.48, 7.94)	5.69 (4.95, 7.31)	0.034
PLT 10^∧^9/L	208.50 (173.00, 243.75)	207.50 (170.75, 241.50)	210.00 (185.00, 244.50)	0.697
Hb g/L	143.00 (132.25, 153.00)	145.00 (130.75, 154.00)	142.00 (132.75, 150.00)	0.668
Lymphocyte	1.94 (1.55, 2.36)	1.87 (1.52, 2.17)	2.04 (1.60, 2.44)	0.199
Neutrophil	3.60 (2.92, 4.59)	3.81 (2.93, 5.08)	3.46 (2.81, 4.09)	0.083
Monocyte	0.37 (0.30, 0.45)	0.39 (0.33, 0.49)	0.34 (0.29, 0.42)	0.005
NLR	1.91 (1.44, 2.51)	2.15 (1.51, 2.72)	1.71 (1.32, 2.12)	0.007
PLR	106.99 (84.10, 131.98)	107.56 (87.43, 143.64)	106.87 (82.75, 126.47)	0.372
SII	400.80 (268.58, 526.69)	430.18 (297.90, 610.85)	356.91 (263.12, 471.54)	0.034
MLR	0.18 (0.16, 0.25)	0.20 (0.18, 0.27)	0.17 (0.15, 0.20)	< 0.001
SIRI	0.69 (0.49, 1.01)	0.86 (0.56, 1.21)	0.57 (0.44, 0.80)	< 0.001
Albumin g/L	47.20 (45.60, 48.98)	46.85 (45.40, 48.90)	47.80 (45.90, 49.23)	0.132
ORM1 μg/mL	35.32 (34.21, 37.36)	37.36 (34.22, 38.35)	35.09 (34.18, 35.93)	< 0.001
ORM2 μg/mL	14.19 (13.17, 15.43)	14.59 (13.69, 16.58)	13.21 (12.19, 14.46)	< 0.001
ORM1_ORM2	2.55 (2.30, 2.80)	2.47 (2.20, 2.72)	2.60 (2.35, 2.89)	0.001
AGP mg/dL	167.75 (150.02, 183.47)	179.67 (152.43, 200.33)	164.22 (145.29, 172.49)	< 0.001

In terms of laboratory indicators, there was a significant difference in the inflammatory levels between the two groups. Although there were no significant differences in the levels of Hb, NEU, LYM, PLT and albumin between the two groups (*p* > 0.05), the WBC and MONO levels of patients with psoriasis were significantly higher than those of the healthy control group (*p* < 0.01). Most notably, the candidate serum biomarkers (ORM1, ORM2, AGP) and the vast majority of systemic inflammation composite indices (NLR, MLR, SII, SIRI) showed a highly significant upward trend in the study group (all *p* < 0.001), suggesting that these indicators are more sensitive in reflecting systemic inflammation of psoriasis compared to conventional blood indicators.

### 3.2 Feature variable selection

This study conducted a single-factor Logistic regression analysis to identify the risk factors for the occurrence of psoriasis in patients (Table 2). Multiple inflammatory and immune-related indicators are significantly associated with the risk of developing psoriasis. The analysis revealed that monocyte count (OR = 1.746, 95% CI: 1.153–3.801, *p* = 0.011), ORM1 (OR = 1.960, 95% CI: 1.515–2.535, *p* < 0.001), ORM2 (OR = 1.689, 95% CI: 1.371–2.081, *p* < 0.001), MLR (OR = 1.940, 95% CI: 1.207–3.291, *p* = 0.012), and AGP (OR = 1.017, 95% CI: 1.006–1.028, *p* = 0.002) are risk factors for the occurrence of psoriasis. It is notable that the interaction term between ORM1 and ORM2 (ORM1_ORM2) shows a strong statistical significance (OR = 0.143, 95% CI: 0.048–0.430, *p* < 0.001), suggesting that the two may jointly act on the disease risk, with an effect that is not simply the simple addition of independent factors. Additionally, variables such as WBC, age, gender, platelet count, hemoglobin, lymphocyte count, neutrophil count, NLR, PLR, SII, SIRI, albumin level, as well as history of alcohol consumption, smoking, hypertension, and diabetes, did not show significant associations with the onset of psoriasis in this study model (all *p* > 0.05).

**TABLE 2:** Risk factors for the occurrence of psoriasis in patients.

Variables	OR (95%CI)	*P*-value
Age, years	1.002 (0.979, 1.025)	0.881
Gender, n(%)		
Female	Reference	
Male	0.665 (0.340, 1.302)	0.234
WBC 10^∧^9/L	1.212 (1.000, 1.468)	0.050
PLT 10^∧^9/L	0.999 (0.993, 1.006)	0.810
Hb g/L	1.000 (0.980, 1.019)	0.972
Lymphocyte	0.671 (0.363, 1.240)	0.203
Neutrophil	1.188 (0.946, 1.493)	0.138
Monocyte	1.746 (1.153, 3.801)	0.011
NLR	1.422 (0.980, 2.065)	0.064
PLR	1.005 (0.995, 1.014)	0.343
SII	1.001 (1.000, 1.003)	0.100
MLR	1.940 (1.207, 3.291)	0.012
SIRI	1.659 (0.935, 2.942)	0.083
Albumin g/L	0.892 (0.776, 1.025)	0.108
ORM1 μg/mL	1.960 (1.515, 2.535)	< 0.001
ORM2 μg/mL	1.689 (1.371, 2.081)	< 0.001
ORM1_ORM2	0.143 (0.048, 0.430)	< 0.001
AGP mg/dL	1.017 (1.006, 1.028)	0.002
Alcohol, n(%)		
No	Reference	
Yes	1.074 (0.513, 2.249)	0.850
Smoking, n(%)		
No	Reference	
Yes	1.304 (0.638, 2.669)	0.467
Hypertension, n(%)		
No	Reference	
Yes	0.836 (0.365, 1.918)	0.673 0.293
Diabetes, n(%)		
No	Reference	
Yes	0.563 (0.193, 1.643)	

**FIGURE 1:**
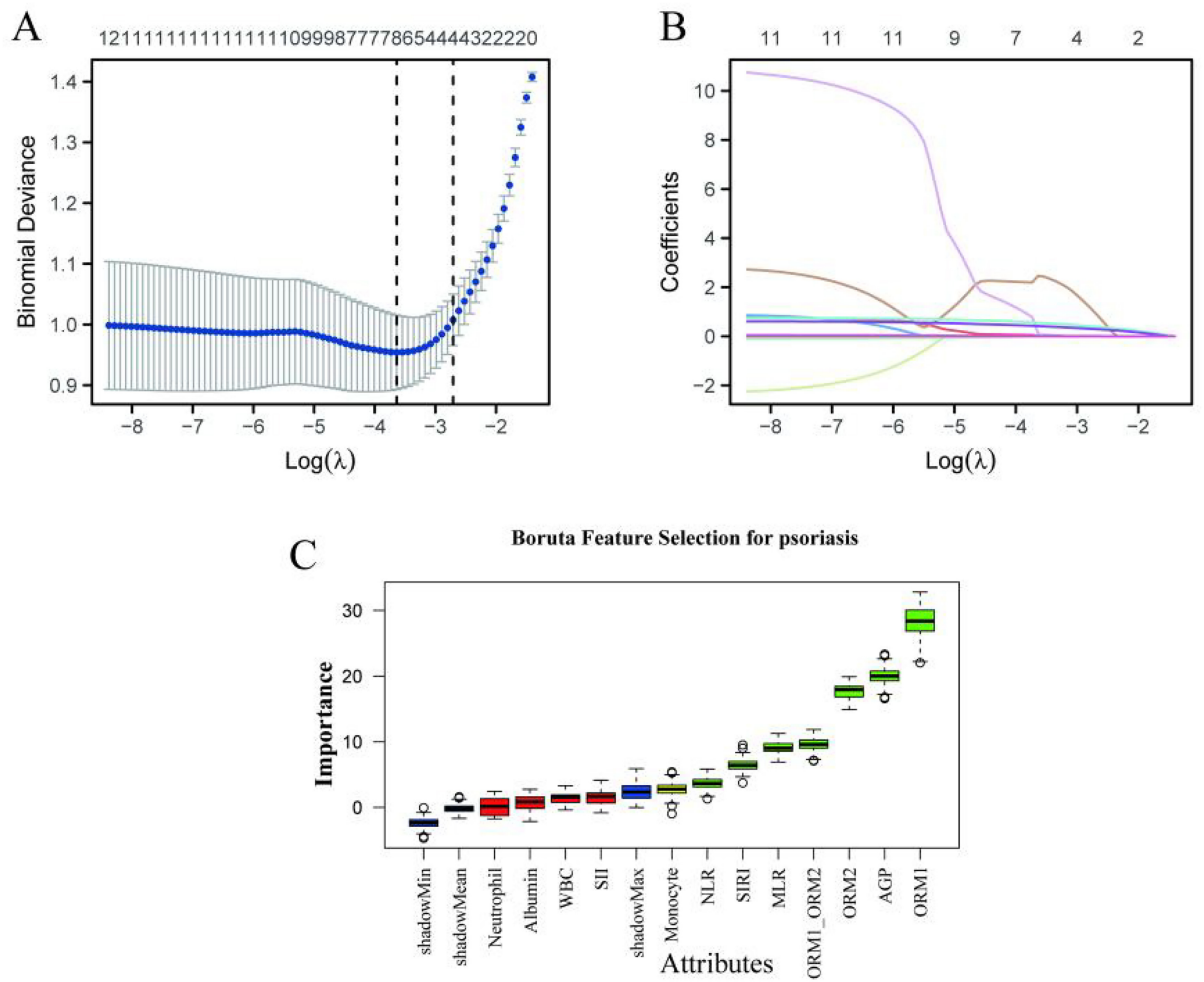
LASSO regression and Boruta algorithm. **(A)** Cross-validation curve for model fitting. **(B)** The contraction path of variable coefficients. **(C)** Boruta Algorithm.

To identify the core biomarkers and avoid the influence of multicollinearity, we further employed LASSO regression and Boruta algorithm to conduct feature selection on the variables with *p* < 0.2 identified in the univariate analysis (Figure 1). LASSO regression analysis achieves variable selection by adjusting the regularization parameter. Figure 1A shows the cross-validation curve of model fitting, where the binomial deviation changes in a trend of first decreasing and then increasing with log(λ), reaching the optimal fit at λ_min. Figure 1B depicts the contraction path of variable coefficients: as log(λ) increases, all coefficients shrink toward zero. At the optimal λ_min, the variable coefficients of MONO, ORM2, ORM1, etc. are non-zero; at the more concise λ_1se, only MONO, ORM2, ORM1, and AGP are retained (with non-zero coefficients), indicating that these features have the strongest predictive robustness. The Boruta algorithm assesses feature importance through random forest (Figure 1C). The results show that the importance scores of variables such as ORM1, ORM2, AGP, MLR, SIRI are significantly higher than those of randomly generated shadow features (green bars), and are confirmed as important predictors; the importance of monocytes is at the borderline level (yellow bars); while variables such as albumin, neutrophil count, white blood cell count, etc. are determined to be unimportant features (red bars).

Based on the results of the single-factor analysis, LASSO regression and Boruta algorithm, we finally selected four key variables, namely MONO, ORM1, ORM2, and AGP, to build the subsequent multi-factor diagnostic model. This selection process considered statistical significance, model simplicity and feature importance, ensuring the biological significance and predictive stability of the selected variables.

### 3.3 The relationship between MONO, ORM1, ORM2, and AGP and psoriasis

**FIGURE 2:**
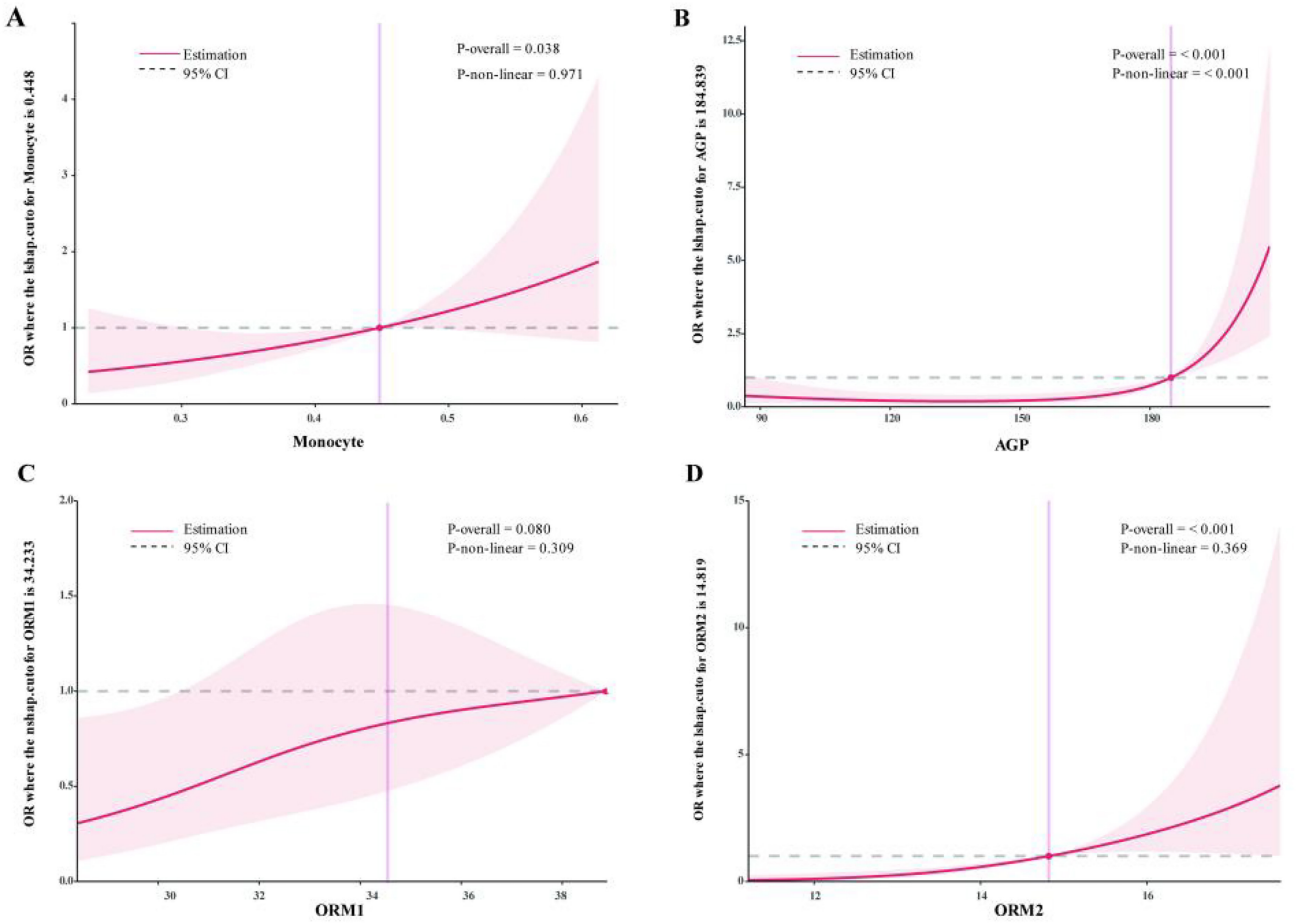
The RCS diagram showing the relationship between key variables and psoriasis. **(A)** MONO. **(B)** AGP. **(C)** ORM1. **(D)** ORM2.

To gain a more intuitive understanding of the relationships between the four key variables (MONO, ORM1, ORM2, and AGP) and psoriasis, this study used non-linear regression analysis to draw an RCS diagram (Figure 2). MONO was positively correlated with the risk of psoriasis, and the overall model was significant (*p* = 0.038), but there was no obvious non-linear relationship (Figure 2A). When the count reached approximately 0.448, the risk significantly increased. AGP was not related to the risk at low concentrations, but the risk sharply increased when it exceeded 184.839. The overall and non-linear relationships were highly significant (*p* < 0.001), indicating that high concentrations of AGP are important risk factors (Figure 2B). ORM1 was slightly positively correlated with the risk, and the overall model was close to being significant (*p* = 0.080), but there was no significant non-linear relationship. The risk changed significantly when the concentration was approximately 34.233 (Figure 2C). ORM2 was not related to the risk at low concentrations, but the risk significantly increased when it exceeded 14.819. The overall and non-linear relationships were significant (*p* < 0.001) (Figure 2D). In summary, MONO, AGP, and ORM2 are significantly associated with the risk of psoriasis, especially AGP and ORM2 show strong non-linear effects, providing potential markers for clinical prediction.

### 3.4 Determination of independent risk factors and model construction

**FIGURE 3:**
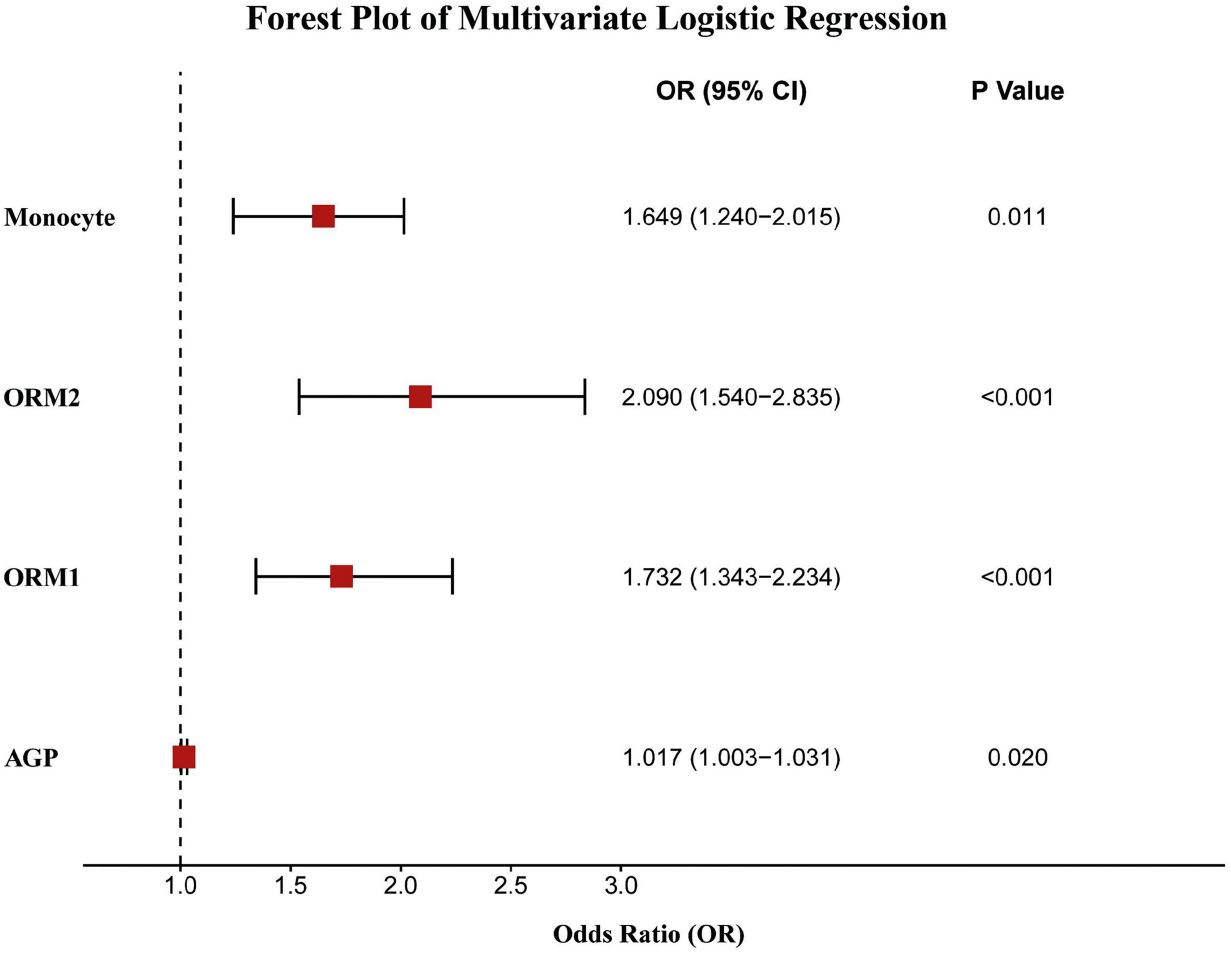
Multivariate logistic regression analysis of independent risk factors for psoriasis.

**FIGURE 4:**
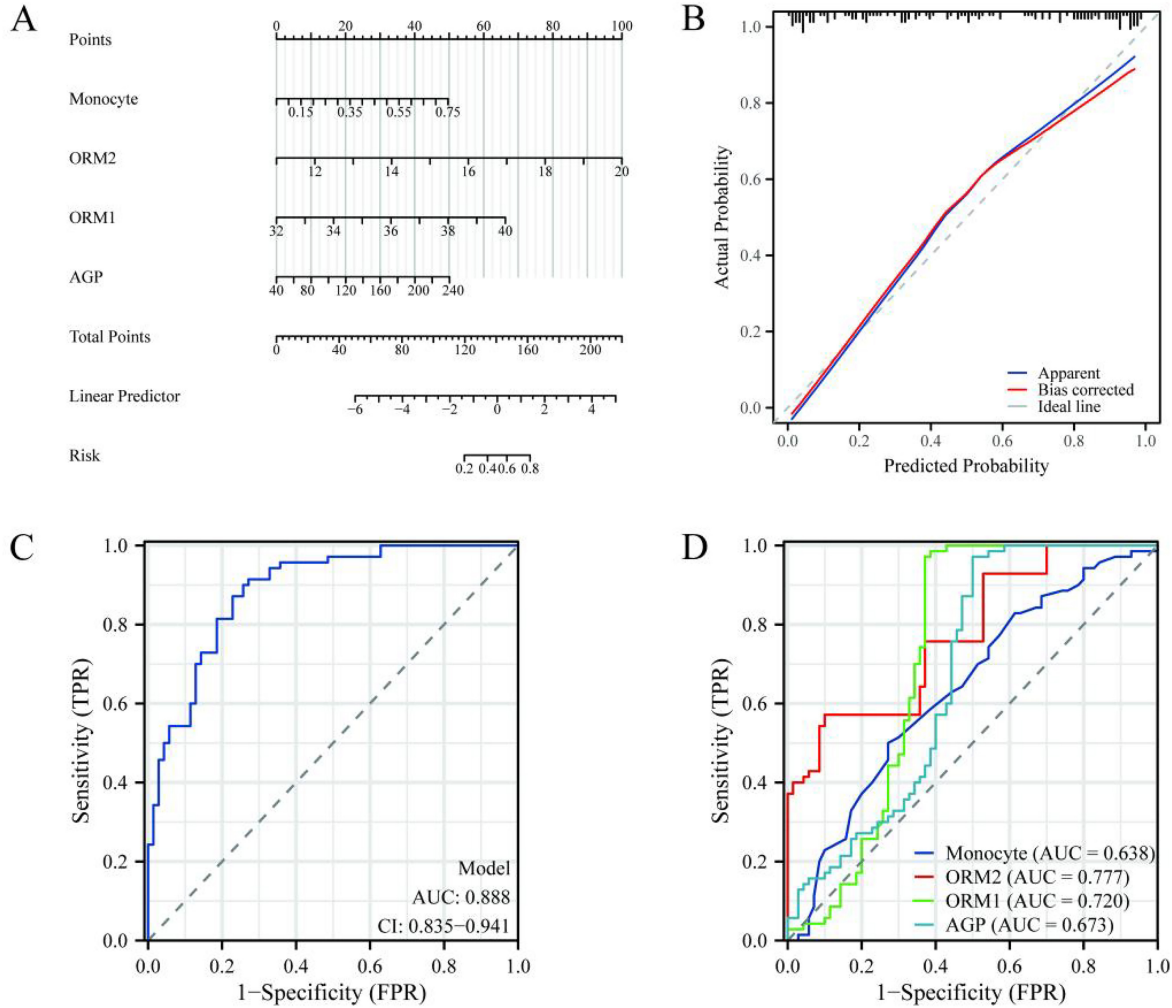
Risk prediction for the onset of psoriasis. **(A)** Nomogram. **(B)** The calibration curve. **(C)** The ROC of the model. **(D)** The ROC curves of each variable.

Through multivariate logistic regression analysis of the four key variables, namely MONO, ORM2, ORM1, and AGP, we determined their status as independent risk factors for psoriasis. The multivariate analysis showed (Figure 3) that MONO (OR = 1.649, 95% CI: 1.240–2.015, *p* = 0.011), ORM2 (OR = 2.090, 95% CI: 1.540–2.835, *p* < 0.001), ORM1 (OR = 1.732, 95% CI: 1.343–2.234, *p* < 0.001), and AGP (OR = 1.017, 95% CI: 1.003–1.031, *p* = 0.020) were all significantly associated with the risk of psoriasis. To further evaluate the predictive value of these variables, we plotted a nomogram (Figure 4A), which transformed the standardized values of each variable into scores. The total score was positively correlated with the risk of psoriasis and was convenient for clinical application. The calibration curve of the model (Figure 4B) showed that the actual probability was highly consistent with the predicted probability, indicating that the model had good calibration performance. The ROC analysis showed (Figure 4C) that the overall predictive ability of the model was excellent, with an AUC of 0.888 (95% CI: 0.835–0.941). Additionally, the ROC curves of each variable analyzed separately (Figure 4D) showed that ORM2 (AUC = 0.777), ORM1 (AUC = 0.720), MONO (AUC = 0.638), and AGP (AUC = 0.673) all had varying degrees of predictive ability for the risk of psoriasis, with ORM2 performing the best.

### 3.5 Differences in the expression of key variables such as MONO, ORM2, ORM1, and AGP between patients with psoriasis and their PASI scores

**FIGURE 5:**
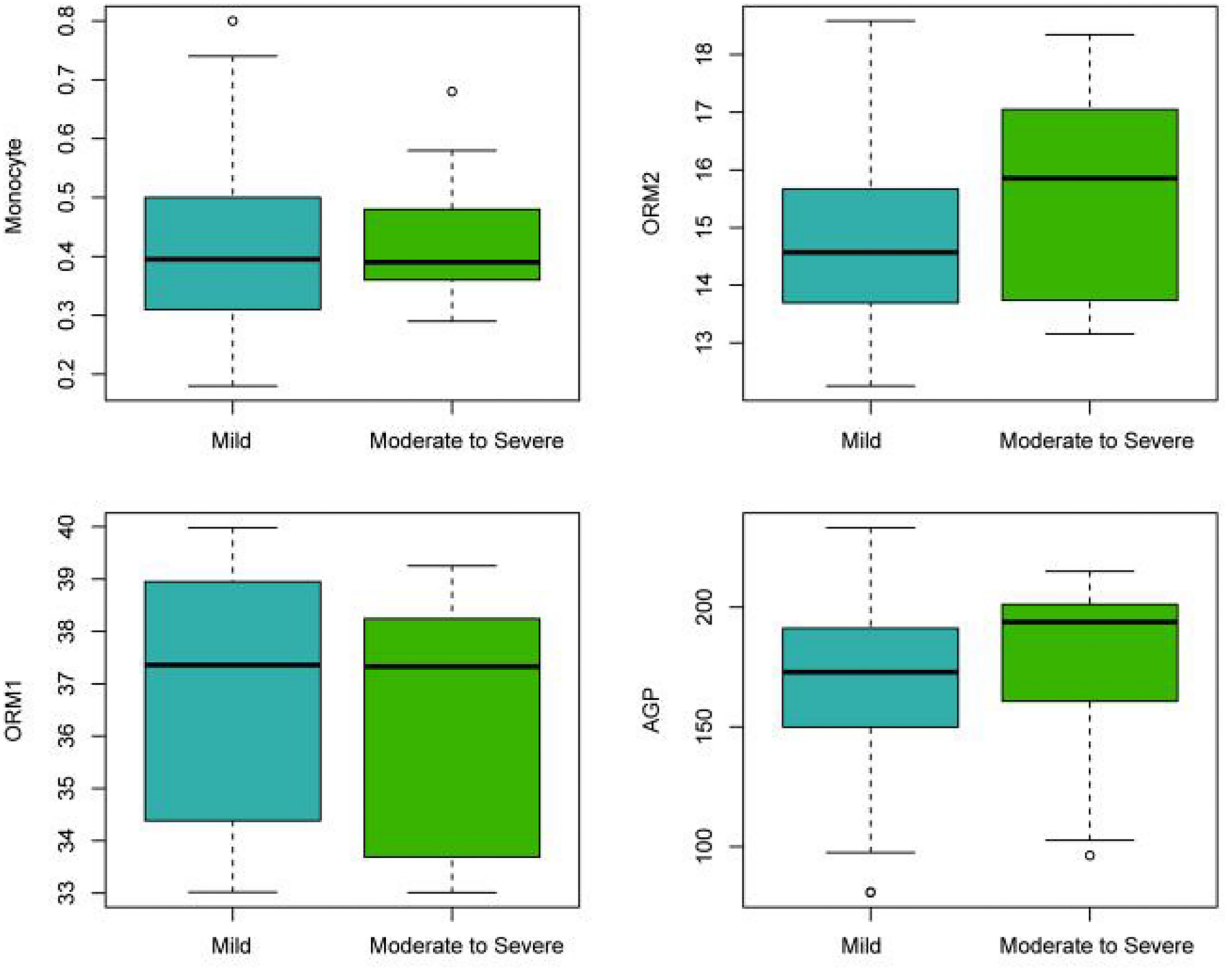
The expression patterns of MONO, ORM2, ORM1, and AGP in different severities of psoriasis.

The expression patterns of MONO, ORM2, ORM1, and AGP in different severity levels of psoriasis (“Mild” and “Moderate to Severe”) (Figure 5) showed some trends despite no statistically significant differences. Specifically, MONO slightly increased in the “Moderate to Severe” group, with the median rising from 0.45 to approximately 0.6; the level of AGP also showed a similar trend, with the median increasing from 170 to approximately 190. The level of ORM2 significantly increased in the “Moderate to Severe” group, with the median rising from 14.5 to approximately 16.5, indicating a more obvious difference. In contrast, the expression of ORM1 was basically consistent between the two groups, with the median being approximately 37.5 in both, showing no significant change.

## 4 Discussion

Psoriasis is a common chronic immune-mediated skin disease. Traditionally, the diagnosis of psoriasis mainly relied on clinical manifestations and skin biopsies. However, due to its heterogeneity, early diagnosis and treatment face many challenges (17). Therefore, researchers began to explore various biomarkers to improve the accuracy and timeliness of diagnosis, thereby providing more effective treatment options for patients. In recent years, biomarkers have gradually gained attention in the research of psoriasis. These markers not only facilitate the early diagnosis of the disease but can also be used to monitor disease progression and evaluate treatment effects. For example, inflammatory factors, long-chain non-coding RNAs, metabolites, and extracellular matrix components are all considered potential diagnostic markers (18, 19). Moreover, with the development of biotechnology, the discovery and clinical application of new biomarkers are constantly advancing, providing new possibilities for individualized treatment of psoriasis (20). This study, through the research of biomarkers such as MONO, ORM1, ORM2, and AGP, provides a new perspective for the early identification of psoriasis.

MONO is an important component of the immune system, mainly derived from the bone marrow, and possesses various biological characteristics. They circulate in the blood and can rapidly migrate to damaged tissues, participating in local immune responses and inflammatory processes. In the pathological process of psoriasis, the function of MONO is particularly important because they not only secrete multiple cytokines but also clear pathogens and dead cells through phagocytosis (21). In the pathogenesis of psoriasis, MONO participates in the progression of the disease through multiple pathways. Firstly, MONO plays a crucial role in skin damage and inflammatory responses. They release pro-inflammatory cytokines [such as Tumor Necrosis Factor-alpha (TNF-α), Interleukin-6 (IL-6), and Interleukin-1 beta (IL-1β)] to promote the inflammatory response, thereby further exacerbating the skin lesions (22). The quantity, subgroup distribution and functional abnormalities of peripheral blood MONO can serve as predictive indicators for the “future risk of onset” or “early activity” of psoriasis. Flow cytometry analysis revealed that the proportion of peripheral monocytes in patients with psoriasis was significantly higher than that in healthy individuals, and it showed a linear positive correlation with PASI (23). Furthermore, studies have shown that MLR can reflect the inflammatory state within the body and is associated with the prognosis of various diseases. The machine learning team conducted a cohort study and found that MLR had the highest predictive power for the risk of developing psoriasis. Using 22,908 peripheral blood routine data, the comparison of nine algorithms showed that MLR had the highest classification efficacy for “future psoriasis status” (AUC = 0.662), outperforming traditional indicators such as NLR and SII (24).

AGP plays a regulatory role in the inflammatory response, and can influence the immune response by binding to various cytokines and drugs. Additionally, AGP also plays an important role in cell signal transduction, cell adhesion, cell growth and differentiation. Studies have shown that the glycosylation state of AGP is closely related to its biological functions. Abnormal glycosylation may lead to the occurrence of various diseases, including cancer and autoimmune diseases (25). The serum AGP concentration in patients with psoriasis was significantly higher than that in healthy controls, and it was negatively correlated with High-Density Lipoprotein Cholesterol (HDL-C), suggesting that it can serve as a window indicator for “systemic inflammation + metabolic risk”; however, no linear correlation was found with PASI at present, so its predictive value for the severity of skin lesions is limited (8). *In vitro* experiments have shown that the acute-phase concentration of AGP can bind to the membrane of monocytes, down-regulate the expression of Cluster of Differentiation 18(CD18), inhibit its chemotaxis and endothelial adhesion, thereby blocking the migration of monocytes from the blood to the tissues (26).

ORM1 and ORM2 both belong to the acute-phase glycoprotein family. Their expressions change in various physiological and pathological conditions, especially in chronic inflammation and autoimmune diseases (27). Under the stimulation of TNF-α/IL-1β, hepatocytes activate the transcription of ORM1/ORM2 genes. The ORM proteins can exert a “negative feedback” anti-inflammatory effect by down-regulating the CD18 integrin and inhibiting the migration and adhesion of monocytes to the endothelium (28). Early case-control studies have shown that the ORM1 allele is significantly enriched in male patients with psoriasis, with a relative risk of approximately 1.9, suggesting that this genotype may affect the susceptibility to the disease or the clinical course (29). The research indicates that urinary ORM levels rise in a stepwise manner along with PASI, and have the potential to serve as a monitoring indicator for “inflammatory activity.” Further large-sample and long-term follow-up studies are needed to clarify its sensitivity/specificity for predicting the occurrence of new psoriasis in the future (10).

This study focuses on the inflammatory indicators and biomarkers of patients with psoriasis. Our predictive model incorporates four indicators: MONO, ORM1, ORM2, and AGP. The calibration curve of the model shows that the actual probability is highly consistent with the predicted probability, indicating that the model has good calibration performance. The overall predictive ability of the model is excellent, with an AUC of 0.888 (95% CI: 0.835–0.941). Additionally, the ROC curves of each variable analyzed separately show that ORM2 (AUC = 0.777), ORM1 (AUC = 0.720), monocytes (AUC = 0.638) and AGP (AUC = 0.673) all have varying degrees of predictive ability for the risk of psoriasis, among which ORM2 performs the best.

However, we must also openly discuss the limitations of this study. Firstly, the sample size is relatively small, and it was conducted in only one center, which may affect the generalizability of the results. Secondly, we lack long-term clinical validation data, which limits our understanding of the long-term efficacy of the biomarkers. Future research can further validate and improve the application of these biomarkers in the management of psoriasis by expanding the sample size and adopting a multi-center design.

In conclusion, this study has revealed significant increases in multiple biomarkers in patients with psoriasis, and has confirmed their potential as independent risk factors. In the future research on psoriasis, more attention should be paid to the combined application of MONO, ORM1, ORM2, and AGP and their potential clinical significance. With a deeper understanding of the interaction mechanisms of these biomarkers, new ideas for individualized treatment of psoriasis may be provided.

## Data Availability

The raw data supporting the conclusions of this article will be made available by the authors, without undue reservation.
